# Sex differences in the inflammatory response of the mouse DRG and its connection to pain in experimental autoimmune encephalomyelitis

**DOI:** 10.1038/s41598-022-25295-y

**Published:** 2022-12-05

**Authors:** Aislinn D. Maguire, Timothy N. Friedman, Dania N. Villarreal Andrade, Fajr Haq, Jacob Dunn, Keiana Pfeifle, Gustavo Tenorio, Karen Buro, Jason R. Plemel, Bradley J. Kerr

**Affiliations:** 1 Neuroscience and Mental Health Institute, University of Alberta, Edmonton, AB T6G 2E1 Canada; 2 Department of Pharmacology, University of Alberta, Edmonton, AB T6E 2H7 Canada; 3 Department of Mathematics and Statistics, MacEwan University, Edmonton, AB T5J 2P2 Canada; 4 Department of Anesthesiology and Pain Medicine, University of Alberta, Clinical Sciences Building, 2-150, Edmonton, AB T6G 2G3 Canada

**Keywords:** Immunology, Neuroscience

## Abstract

Multiple Sclerosis (MS) is an autoimmune disease with notable sex differences. Women are not only more likely to develop MS but are also more likely than men to experience neuropathic pain in the disease. It has been postulated that neuropathic pain in MS can originate in the peripheral nervous system at the level of the dorsal root ganglia (DRG), which houses primary pain sensing neurons (nociceptors). These nociceptors become hyperexcitable in response to inflammation, leading to peripheral sensitization and eventually central sensitization, which maintains pain long-term. The mouse model experimental autoimmune encephalomyelitis (EAE) is a good model for human MS as it replicates classic MS symptoms including pain. Using EAE mice as well as naïve primary mouse DRG neurons cultured in vitro, we sought to characterize sex differences, specifically in peripheral sensory neurons. We found sex differences in the inflammatory profile of the EAE DRG, and in the TNFα downstream signaling pathways activated intracellularly in cultured nociceptors. We also found increased cell death with TNFα treatment. Given that TNFα signaling has been shown to initiate intrinsic apoptosis through mitochondrial disruption, this led us to investigate sex differences in the mitochondria’s response to TNFα. Our results demonstrate that male sensory neurons are more sensitive to mitochondrial stress, making them prone to neuronal injury. In contrast, female sensory neurons appear to be more resistant to mitochondrial stress and exhibit an inflammatory and regenerative phenotype that may underlie greater nociceptor hyperexcitability and pain. Understanding these sex differences at the level of the primary sensory neuron is an important first step in our eventual goal of developing sex-specific treatments to halt pain development in the periphery before central sensitization is established.

## Introduction

Multiple sclerosis (MS) is an autoimmune disease characterized by widespread inflammation, immune cell activation, and demyelinating lesions of the central nervous system (CNS)^1^. The symptoms of MS are extensive, affecting sensory, motor, and cognitive functions^2,3^. One of the most debilitating symptoms, which affects over half of patients, is pain^4^. Adding another level of complexity to this condition, women are more likely than men to experience pain in MS^4–6^. Neuropathic pain is caused by injury or disease of the nervous system, producing pain in response to non-noxious stimuli (allodynia), increased pain in response to noxious stimuli (hyperalgesia), and spontaneous pain which occurs without a stimulus. Neuropathic pain can originate in the peripheral nervous system (PNS) within the dorsal root ganglia (DRG), which house the cell bodies of primary pain-sensing neurons called nociceptors^7,8^. The hypothesis for MS pain, developed in animal research, is that inflammation and immune cell activation in the DRG cause nociceptors to become hyperexcitable, meaning they respond more readily and intensely to painful stimuli^9,10^. Peripheral sensitization of nociceptors triggers similar mechanisms in the spinal cord and brain, leading to central sensitization which maintains pain persistently, regardless of disease progression^7,8^. This may help explain why conventional pain treatments are ineffective in the treatment of neuropathic pain in MS^4,11^. While we know from previous work in our lab that there are sex differences in nociceptor hyperexcitability in an MS animal model^12^, the underlying mechanism is unknown. Before we can speculate on pain mechanisms in human MS, we must investigate the DRG more extensively in animal models.

To further examine the mechanisms of peripheral sensitization and uncover potential sex differences, we used the animal model, experimental autoimmune encephalomyelitis (EAE)^13,14^. Not only does EAE replicate the inflammation and demyelination found in MS, but most importantly for our purposes, EAE animals exhibit classic signs of neuropathic pain^15–18^. We also used a simplified cell culture model to specifically study naïve primary DRG neurons exposed to the inflammatory cytokine TNFα. We chose TNFα as our stimulus because it is upregulated both peripherally and centrally in MS and EAE, it causes sensory neuron hyperexcitability, and it is involved in the development of many neuropathic pain conditions, and it is link^19–23^. Past work from our lab has also shown upregulation of TNFα from circulating immune cells in EAE^12^. These findings informed the concentration of TNFα we selected (1 ng/mL) as we aimed to use a physiologically relevant dose. A major focus of this study was on mitochondrial responses to TNFα between the sexes. Mitochondrial dysfunction has been linked to neuropathic pain, and is known to be regulated by TNFα effectors, c-jun N-terminal kinase (JNK) and P38 mitogen-activated protein kinases (MAPKs), as well as nuclear factor κB (NFκB)^24–27^. In this study, we reveal sex differences in the inflammatory response to disease, TNFα signaling, mitochondrial function, neuronal injury, and plasticity in the DRG in vivo and in vitro*.* These differences suggest that males and females engage distinct, sex-specific pathways at the level of the DRG that may have important implications on the development of neuropathic pain in EAE.

## Materials and methods

### Experimental autoimmune encephalomyelitis

As previously described^28^, we induced EAE in both male and female C57BL/6 mice (5 animals per group, 8–10 weeks old, Charles River) by subcutaneous injection of 50 μg of myelin oligodendrocyte glycoprotein (MOG_35-55_) emulsified in complete Freund’s adjuvant (CFA, 1.5 mg/mL). On the same day as induction, and 48 h later, mice were also inoculated with 300 ng of pertussis toxin. Animals were euthanized and perfused with cold saline on the first day of EAE symptom onset alongside their “CFA controls”, which received only the adjuvants, CFA and Pertussis, but not MOG_35-55_. Tissue was dissected and snap frozen in liquid nitrogen, then stored at − 80 °C. All animal experiments were performed according to the Canadian Council on Animal Care's Guidelines and Policies with approval from the University of Alberta Health Sciences Animal Care and Use Committee (protocol 0,000,274). This study is reported in accordance with ARRIVE guidelines.

### Immunohistochemistry (IHC)

Fresh frozen DRG tissue was cryo-sectioned at 10 μm thickness. Tissue slides were then immersed in 4% paraformaldehyde (PFA) and fixed for 5 min at room temperature. Next, they were immersed in antigen retrieval solution (1.92 g anhydrous citric acid and 0.5 mL Tween20 in 1 L H_2_O, pH 6.0) for 10 min before 3 × 10-min washes in PBS. Tissue was blocked for one hour at room temperature in 10% normal donkey serum (NDS) in 0.2% triton X-100 in PBS (PBS_TX_). Primary antibody was incubated overnight at 4 °C in 2% NGS and 2% bovine serum albumin (BSA) in PBS_TX_. Primary antibodies included CD45 (1:200, BD Pharmigen 550539), Iba1 (1:500 Cellular Dynamics 019-19741), CD3 (1:200, Bio-Rad MCA1477), cleaved caspase 3 (1:100, Cell Signaling #9661), ATF3 (1:200, Abcam ab207434), and pCREB (1:500, Cell Signaling #9198).

**Figure 1:**
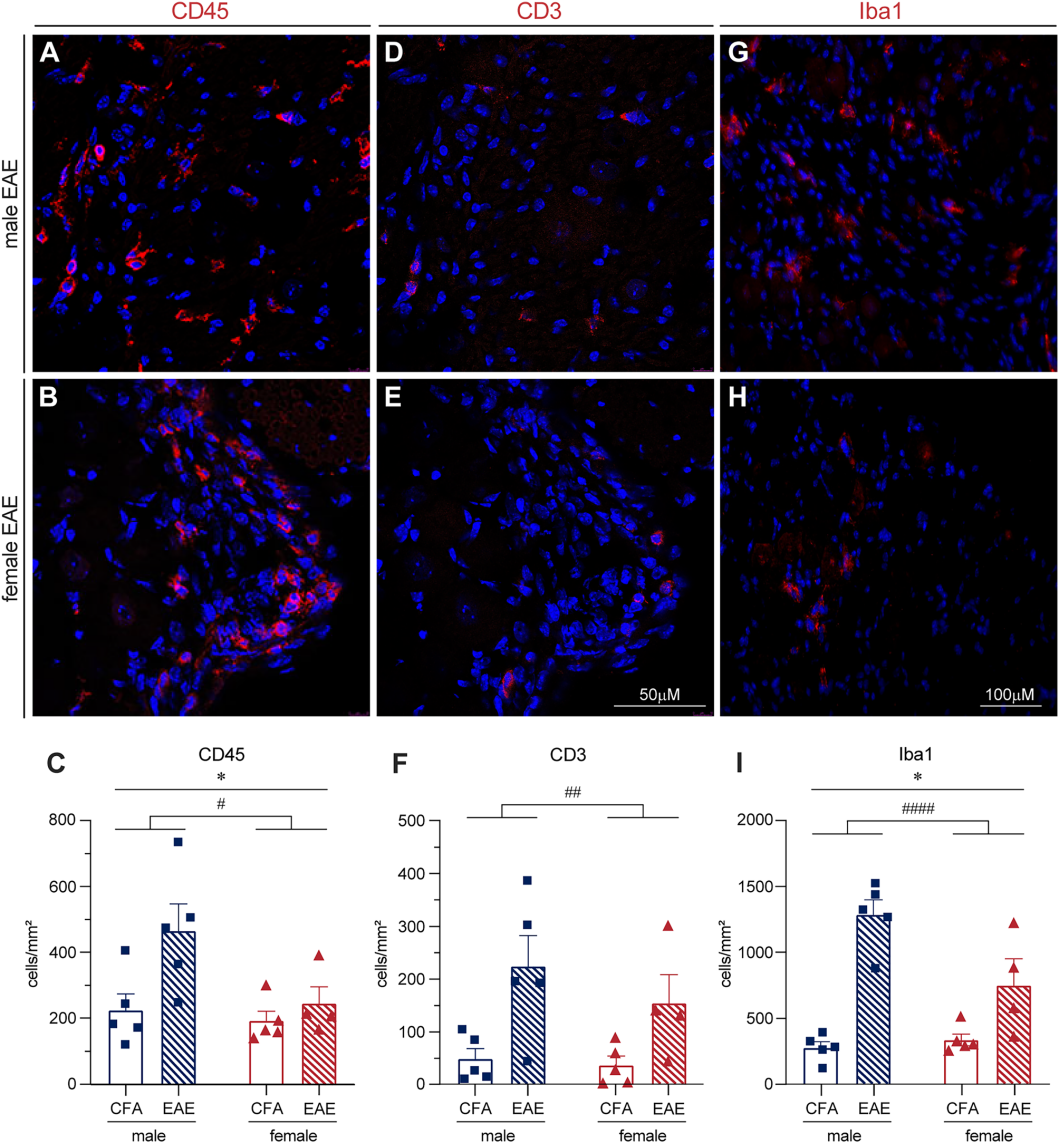
Increased immune cell staining in mouse EAE DRGs in vivo. Lumbar DRG tissue was collected from male and female EAE mice at disease onset, as well as from their respective control mice (referred to here as CFA). CD45 (**A–C**), CD3 (**D–F**), and Iba1 (**G–I**) staining increases in both sexes at EAE onset compared to CFA controls. CD45 and Iba1 staining exhibit a greater increase in male DRGs than females. Bars indicate mean (positive cells/DRG area) ± standard error mean (SEM) n = 5 animals over one experiment. #p < 0.05, ##P < 0.01, ####P < 0.0001 treatment factor, *P < 0.05 sex factor. Two-way ANOVA.

Slides were washed the next day 3 × 10 min in PBS before incubation with secondary antibodies (Jackson Immunoresearch) at a 1:200 dilution for 45 min. Slides were again washed 3 × 10 min in PBS before mounting with fluoromount G™ Mounting Medium (Invitrogen 00-4958-02). Slides were imaged at 20 × for analysis using a Zeiss Axio Observer Z1. Representative images in Fig. 1A,B,D,E were taken at 40 × using a Leica TCS SPE Confocal.

### Immunopanned primary DRG cultures

Immunopanning protocol was adapted from Zuchero 2014^29^. All DRGs were collected from 2–4 male and 2–4 female 8–10 week old C57BL/6 mice, and digested with 2 mg/mL Stemxyme I (Worthington LS004106) for 1 h at 37 °C. Cells were triturated gently and spun for 10 min at 300 × g. They were next resuspended and strained through a 70 μm filter then spun through a 3 mL 15% BSA cushion at 300 xg to remove myelin debris. After the BSA cushion, cells were allowed to rest for 30 min in a 10% CO_2_ incubator at 37 °C for antigen retrieval.

Immunopanning dishes were coated with secondary anti-rat, rabbit, and mouse antibodies (1:500, Jackson 112-005-167, 111-005-003, and 115-005-020 respectively) overnight at 4 °C, then washed and coated with rat CD45 (10 μg, BD Pharmigen 550539), rabbit PDGFRβ (10 μg, Abcam ab32570), and mouse O4 (1:2.5, O4 hybridoma^30,31^) primary antibodies for 2 h at room temperature. These antibodies were intended to deplete immune cells, fibroblasts, and schwann cells respectively. Cells were panned in 5 mL of panning buffer (0.02% BSA and 0.008% DNAse in D-PBS) in each of the 3 dishes for 20 min, shaking gently at 10 min. Cells were spun down, counted with a hemocytometer, and pre-plated for 30 min in 25 μL of panning buffer prior to flooding wells with media (1:1 DMEM:Neurobasal, supplemented with SATO, N-acetyl-cysteine, insulin, penicillin/streptomycin, B27 + , glutamate, and sodium pyruvate). Cells for protein collection (ELISA and Western Blotting) were plated in 24 well clear plastic plates (Falcon 353047) at 2000 cells per well. Cells for imaging were plated in 24 or 96 well black glass-bottom plates (Cellvis P24-1.5H-N, and Falcon 353219 respectively) at 500 cells per well. Cells were allowed to rest 24 h in culture before treatment with 1 ng/mL TNFα for 1, 6, or 24 h. P38 inhibition was performed on cultures treated with 1 ng/mL TNFα, and either 0, 1, or 25 μM of the inhibitor SB203580 (Tocris 1402/10).

### Western blotting

Protein lysates from cultured cells were scraped collected with RIPA lysis buffer (Thermofisher 89900) with cOmplete EDTA-free, (Roche 04693159001) and PhoSTOP (Roche 04906837001) added. Lysates were stored at − 80 °C. Prior to blotting, protein was precipitated with 4 volumes of cold acetone for one hour at 4 °C, then centrifuged a 10,000×*g* for 10 min before resuspension in a smaller volume. Lysates were then diluted with 4 × Bio-Rad laemmli sample buffer and 50 mM dithiothreitol and boiled for 10 min at 95 °C.

Samples were loaded into 4–20% Mini-PROTEAN TGX Stain-free gels (Bio-Rad 4568096), and run at 120 mV for 1–1.5 h. After running, the total protein stain in the gels was activated using the Bio-Rad ChemiDoc XRS + . Gels were then transferred onto low-fluorescence PVDF membranes (Bio-Rad BioRad 1620264) using extra thick blot paper (Bio-Rad 1703965) on the Bio-Rad Trans-Blot Turbo system V1.02 for 30 min at 25 V and 1.0 A. Total protein was then imaged with the ChemiDoc.

Membranes were blocked for one hour at room temperature in 5% BSA dissolved in 0.5% TBS-T. Primary antibody was incubated overnight at 4 °C in 1% BSA in TBS-T. Primary antibodies included phospho-P65 (1:500, Millipore MAB3026), P38 (1:500, Cell Signaling #8690S), phospho-P38 (1:500, Cell Signaling #4631S), JNK (1:500, Cell Signaling #9252S), and phospho-JNK (1:500, Cell Signaling #4668S). Membranes were washed 3 × 10 min in TBS-T before incubation with secondary antibody, Goat anti-Rabbit HRP (1:10 000, Jackson Laboratories 111-035-144) for 1 hour at room temperature. Membranes were again washed 3 × 10 min in TBS-T, then for 10 min in TBS before imaging with the ChemiDoc after 5 min incubation with ECL Prime (Amersham RPN2232). Membrane stripping was performed according to the mild stripping protocol from Abcam.

### Live staining

For mitochondrial morphological characterization, live cells were stained with MitoTracker™ Deep Red FM at 1:10 000 for 30 min, and NucBlue™ Live ReadyProbes™ reagent at 1:200 for 10 min. Cells were then fixed in 4% PFA for 15 min at room temperature and washed with PBS before imaging. Footprint analysis was performed with Fiji MiNA software from the Stuart lab at Brock University^32^. Morphology analysis was adapted from Leabeau et al*.* 2018^33^.

Mitochondrial superoxide was stained for with MitoSOX™ red dye (Invitrogen M36008) at 1:1000 concentration and NucBlue™ Live ReadyProbe (1:200 Invitrogen R37605). Cells were imaged live in an environment controlled Molecular Devices ImageXpress Micro high content screening system.

Cell death was assessed using Propidium Iodide (PI) (1:20 Invitrogen P3566) labelling and NucBlue™ Live ReadyProbe (1:200 Invitrogen R37605). Cells were imaged live in an environment controlled Molecular Devices ImageXpress Micro high content screening system.

### Immunocytochemistry

Cells were fixed in 4% PFA for 15 min at room temperature, then washed 3 × with PBS and blocked in 10% NDS in PBS_TX_. Cleaved caspase 3 primary antibody (1:250, Cell Signaling #9661) was diluted in 2% NGS and 2% BSA in PBS_TX_ at 4 °C overnight. Cells were washed 3 × in D-PBS before incubation with secondary antibody (1:500 goat ant-rabbit 488, Invitrogen A11008) and DAPI (1:2000, Invitrogen D1306) at room temperature for 30 min. Cells were imaged in a Molecular Devices ImageXpress Micro high content screening system.

### Cytochrome C ELISA

Cytochrome C ELISA was performed on cell lysates from immunopanned cultures described in Sect. “Immunopanned primary DRG cultures”, which were scraped and collected in 0.5% PBS_TX_. The protocol for the kit (R&D systems MCTC0) was followed with no variations.

### Data analysis and experimental design

Immunohistochemistry data in Fig. 1 represents cells positive for the stain of interest (counted manually) normalized to tissue area (cells/mm^2^). Each data point represents one animal (n = 5), and was calculated from at least two sections from different DRGs. Analyzed by regular two-way ANOVA (significance was set at P < 0.05).

Western blots were analyzed using Bio Rad Image Lab 6.0.1 software. The majority of the stained area in each lane was quantified for the total protein stain, using boxes of the same dimensions for each lane. Data for P38 and JNK represents the intensity of phosphorylated antibody over total antibody staining, normalized to total protein. NFκB data represents P65 antibody staining normalized to total protein due to lack of a sufficient total P65 antibody. Each data point represents one experiment. DRGs were pooled from between 2–4 naïve animals, and after immunopanning cells were plated at 2000 cells/ well in three replicate wells per group. Lysates from replicate wells were pooled before western blotting. Graphical data was expressed as a fold change of sex-respective unstimulated controls. Analyzed by regular two-way ANOVA (significance was set at P < 0.05).

Mitochondrial footprint data represents the total area of mitochondrial staining, normalized to cell diameter. Stain area was quantified using the Mitochondrial Network Analysis (MiNA) plug-in for ImageJ developed by Valente et al. 2017^32^. Each data point represents the average footprint from one experiment (n = 4). Graphical data was expressed as a fold change of sex-respective controls. Analyzed by regular two-way ANOVA (significance was set at P < 0.05).

Mitochondrial morphology data represents the average proportion of each mitochondrial network classification (elongated, tubular, and fragmented) across five separate experiments (n = 5). In each experiment, roughly 50 cells were imaged per treatment condition, then scored in one of the three morphology categories as per the method developed by Lebeau et al*.* 2018^33^. Statistics were performed by Dr. Karen Buro of MacEwan University using Fisher exact tests with Bonferroni’s multiple comparison.

Mitochondrial superoxide data was collected from two separate experiments, which consisted of four wells each (n = 8 total across two experiments). 12 non-overlapping images were collected from each well. Images were analyzed using MetaXpress software. Data represents mean integrated intensity of MitoSOX™ staining, and is expressed as a fold change of sex-respective controls. Analyzed by regular two-way ANOVA (significance was set at P < 0.05).

Cytochrome C data was collected from three separate experiments, which consisted of three wells each (n = 9 across three experiments). Each well analyzed by ELISA separately. Data represents cytochrome C concentration in ng/mL expressed as a fold change of sex-respective controls. Analyzed by regular two-way ANOVA (significance was set at P < 0.05).

Cleaved caspase 3 immunocytochemistry data was collected from two separate experiments, which consisted of four wells each (n = 8 total across two experiments). 12 non-overlapping images were collected from each well. Images were analyzed using MetaXpress software. Data represents the percentage of DAPI-positive cells that were positive for cleaved caspase 3 staining, and is graphically expressed as a fold change of sex-respective controls. Analyzed by regular two-way ANOVA (significance was set at P < 0.05).

Immunohistochemistry data in Figs. 6, 7, 8 represents cells positive for the stain of interest (counted manually) normalized to tissue area (cells/mm^2^). Each data point represents one animal (n = 5), and was calculated from at least two sections from different DRGs. Graphical data is expressed as a fold change normalized to sex-respective control. Analyzed by regular two-way ANOVA (significance was set at P < 0.05).

### Ethics approval and consent to participate

All animal experiments were performed according to the Canadian Council on Animal Care’s Guidelines and Policies with approval from the University of Alberta Health Sciences Animal Care and Use Committee (protocol 0000274).

## Results

### Both sexes have increased immune cell marker staining in EAE DRGs, but males have greater CD45 and Iba1 staining than females

Previous work from our lab has revealed inflammation in the DRGs of post-mortem female MS patients^34^, as well as inflammatory cell infiltration in both dorsal root and trigeminal ganglia of female mice with EAE^15,35^. Immune cell infiltration and activation in these ganglia in female EAE mice has also been found by Duffy et al*.* 2016^36^. Here, we sought to compare the inflammatory signature of EAE DRGs in both sexes. Using immunohistochemistry, we found significant increases in staining for the immune cell marker CD45 in both male and female EAE DRGs compared to their CFA controls (Fig. 1A–C,F(1, 15) = 6.676, P = 0.0208, Two-way ANOVA), as well as a sex difference in that there was more CD45 stained cells in male tissue (F(1, 15) = 4.938, P = 0.0421, Two-way ANOVA). When we stained for the T cell marker, CD3, and again saw more staining in the EAE group when compared to CFA controls (Fig. 1D–F,F(1, 15) = 13.21, P = 0.0024, Two-way ANOVA), however we did not find a sex difference. Finally, we also observed an increase in Iba1 staining, indicative of an increased macrophage and monocyte presence, in both male and female EAE DRGS (Fig. 1G–I,F(1, 15) = 47.79, P < 0.0001, Two-way ANOVA). However, there was more Iba1 staining in males than females (F(1, 15) = 5.031, P = 0.0404, Two-way ANOVA).

### There are sex differences in TNFα downstream signaling in vitro

**Figure 2:**
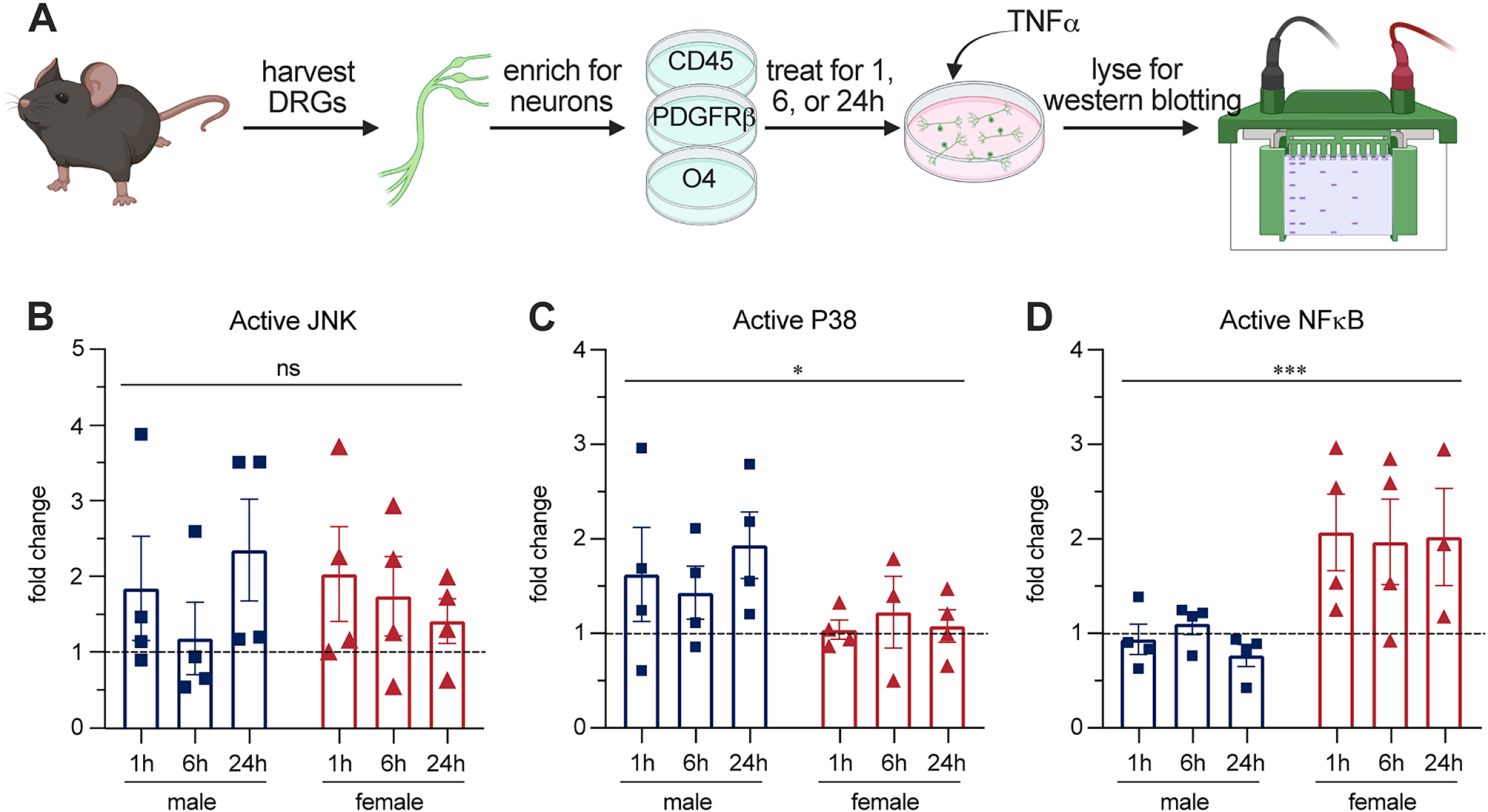
Sex specific activation of the TNFα signaling pathway in vitro. (**A**) DRGs were harvested from male and female mice, immunopanned to enrich the proportion neuronal cells, treated for 1, 6 or 24 h with 1 ng/mL TNFα, then collected for western blotting. (**B**) There were no sex differences evident in JNK phosphorylation. (**C**) Male cells have greater P38 phosphorylation (**D**) Female cells have greater NFκB (P65 subunit) phosphorylation. Bars indicate mean (fold change of active over total protein of interest, normalized to total protein) ± standard error mean (SEM). Dashed line represents untreated condition mean n = 4 experiments. *P < 0.05, ***P < 0.001, sex factor, two-way ANOVA.

TNFα is a pleiotropic cytokine that has been linked to various neuropathic pain conditions and is present both peripherally and centrally in MS^20,37^. Our lab has shown previously that TNFα is increased peripherally in circulating immune cells in EAE^12^. To confirm this result in our current tissue set, we assessed TNFα levels in the male and female EAE DRG by polymerase chain reaction (PCR) using previously described methods^38^. We found TNFα mRNA is elevated in both sexes in EAE (Supplementary Fig. 1). TNFα is secreted by macrophages, monocytes, and lymphocytes, and we know from prior work that there is an increased presence of these inflammatory cell types in the DRG with EAE in females^35^. Additionally, we have confirmed here that these cell types are present in both sexes **(**Fig. 1). For these reasons, we selected TNFα as an in vitro stimulus to understand how it might contribute to sensitization of sensory neurons in the EAE model, and whether it may do so in a sex-specific manner. We selected a physiologically relevant dose of TNFα (1 ng/mL) based on our previous findings^12^. We immunopanned male and female DRGs to reduce the proportion of non-neuronal cells in the culture, then treated them with TNFα for 1, 6, or 24 h. Cells were collected and western blotted (Fig. 2A) for activated (phosphorylated) forms of three main TNFα signaling effectors, JNK, P38, and NFκB to look for sex differences in the TNFα signalling pathway (Supplementary Fig. 2). There was no sex difference evident in JNK activation with TNFα treatment (Fig. 2B,F(1, 20) = 0.01980, P = 0.8895, Two-way ANOVA). However, male sensory neurons exhibited greater levels of p-P38 than females (Fig. 2C,F(1, 19) = 4.900 P = 0.0393, Two-way ANOVA). In contrast, female sensory neurons had higher levels of p-NFκB than males (Fig. 2D,F(1, 19) = 18.55, P = 0.0004, Two-way ANOVA). This led us to investigate the implications these sex differences in TNFα signaling might have further downstream.

### A physiological dose of TNFα causes DRG neuron cell death at 24 h of treatment in vitro.

**Figure 3:**
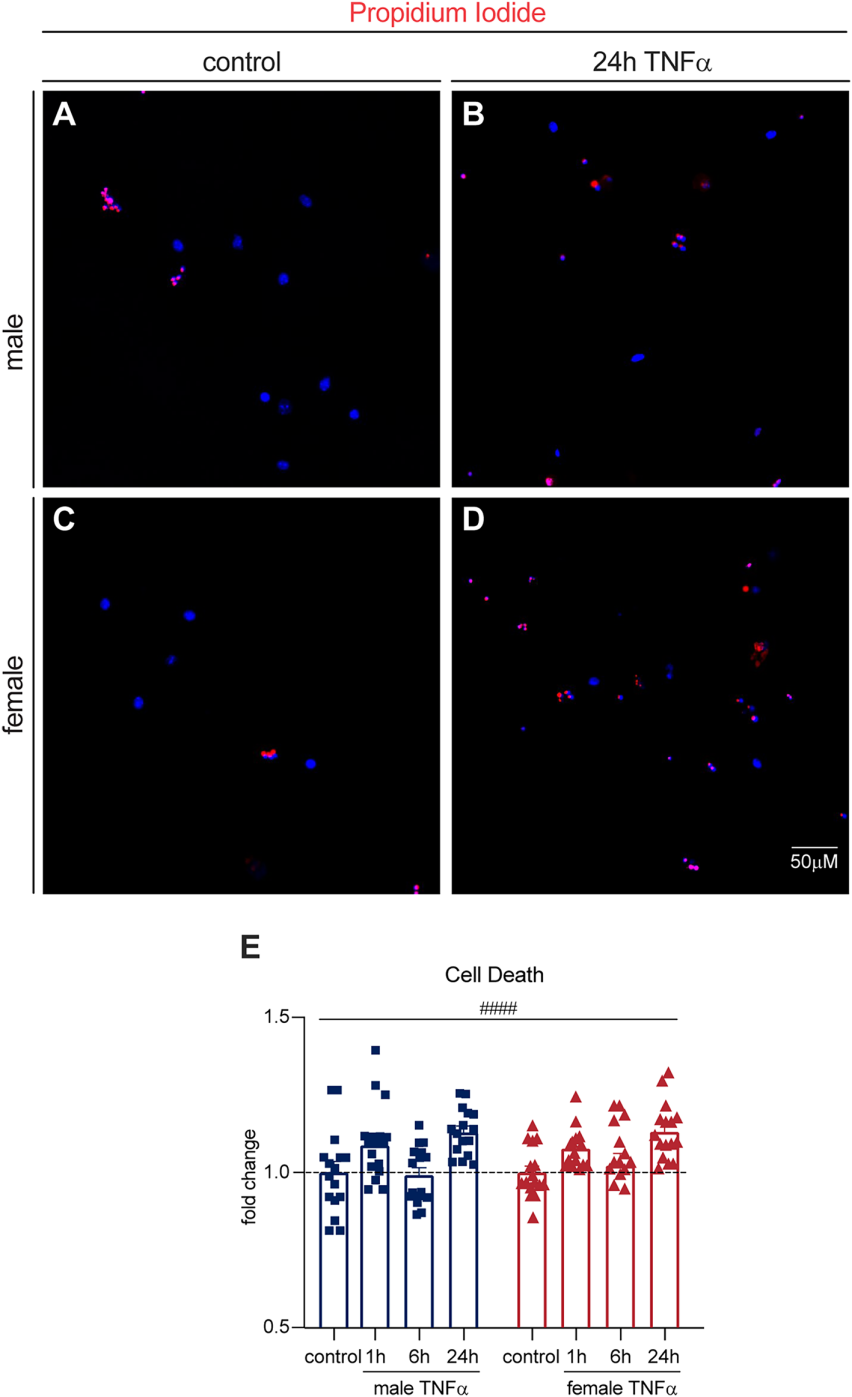
TNFα treatment increases cell death in both sexes in vitro. (**A–D**) Both sexes have increased PI staining with TNFα treatment, particularly at 24 h. (**E**) Bars indicate mean (fold change of PI-positive/total DAPI-positive cells, normalized to untreated controls) ± standard error mean (SEM). Dashed line represents untreated condition mean n = 16 across 4 experiments. ####P < 0.0001, treatment factor, two-way ANOVA.

Neurons express TNF receptor subtype 1 (TNFR1), which is activated by soluble TNFα^39^. This interaction mediates both pro-inflammatory signaling and cell death via either apoptosis or necroptosis^40^. Our naïve DRG neuron cultures were stimulated with soluble TNFα, therefore we investigated cell death. Cultures were stained with PI as a marker of dead cells (Fig. 3A–D). We found a significant increase in PI staining with TNFα treatment (Fig. 3E,F(3, 120) = 11.36, P < 0.0001, Two-way ANOVA). Specifically, at 24 h of TNFα treatment, both males and females had significantly more PI staining than untreated cells (P = 0.0019 and 0.0018 respectively, Dunnett’s multiple comparisons). Intrinsic apoptosis via TNFα signaling is mediated by mitochondrial damage and eventual cytochrome C release^41^. To determine if cell death in response to TNFα was apoptotic in nature, we next assessed mitochondrial structure and function.

### Mitochondrial morphology is affected by TNFα treatment in both sexes in vitro

**Figure 4:**
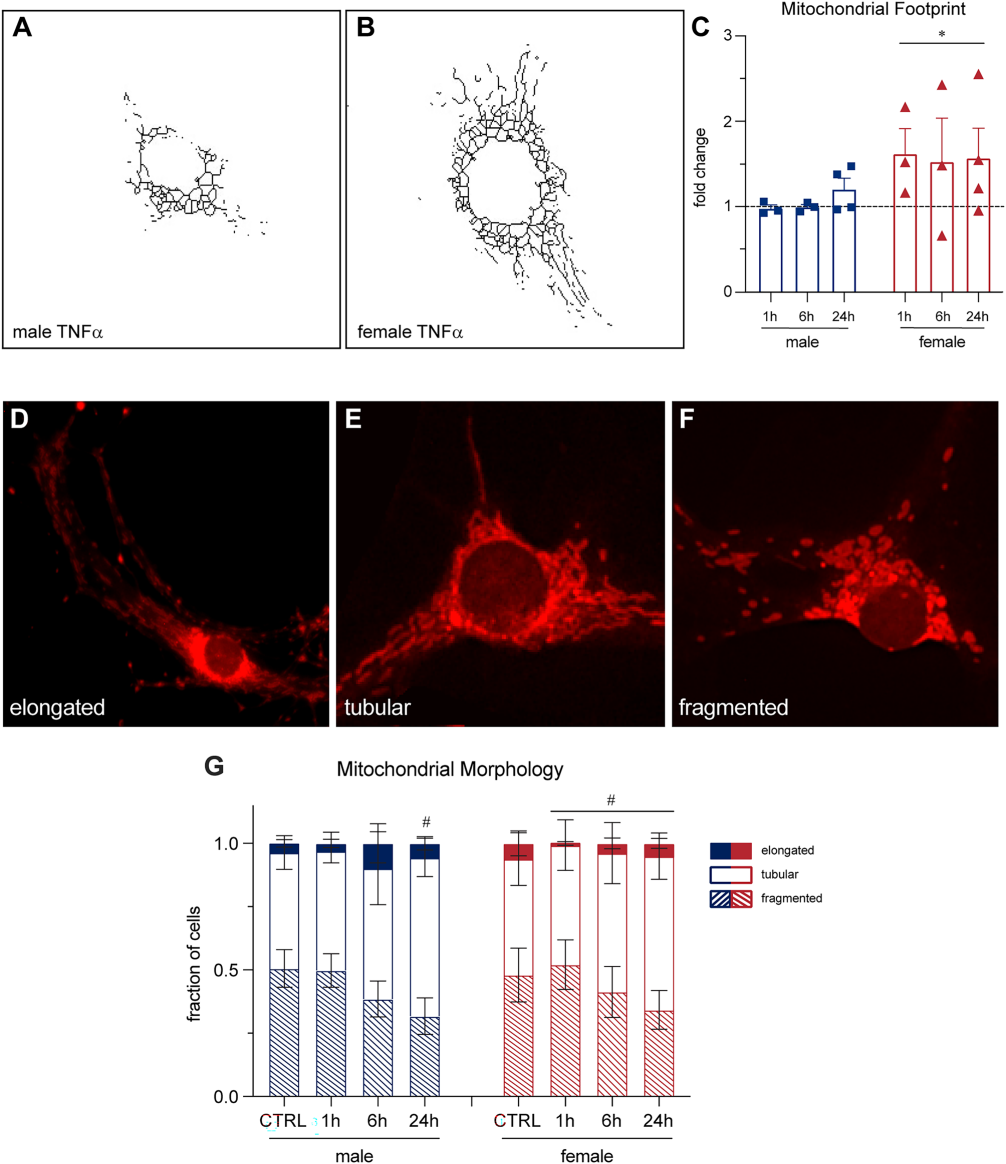
Mitochondrial morphology changes in both sexes in response to TNFα treatment in vitro. (**A,B**) Female cells increased their mitochondrial footprint compared to males with TNFα treatment, determined using Fiji MiNA software. (**C**) Bars indicate mean (fold change of mean footprint/ diameter, normalized to untreated controls) ± standard error mean (SEM). Dashed line represents untreated condition mean n = 4 experiments. *P < 0.05, sex factor, two-way ANOVA. (**D**–**F**) Cells were manually scored into three categories of mitochondrial morphology; elongated, tubular, and fragmented. (**G**) The morphology breakdown changed in males with 24 h of TNFα treatment, and in females with 1, 6 and 24 h of treatment. Bars indicate mean fraction of cells ± standard error mean (SEM) n = 4 experiments. #P < 0.0083, Fisher exact test, Bonferroni’s multiple comparison.

We determined that male sensory neurons may preferentially activate P38 while female neurons may preferentially engage the NFκB pathway in response to TNFα treatment in vitro (Fig. 2). TNFα signaling through both NFκB and P38 has been linked to mitochondrial changes^24–27^, so we next sought to characterize whether exposure to TNFα leads to changes in mitochondrial morphology in male and female nociceptors (DRG neurons ≤ 30 μm in diameter). We first stained for mitochondria and assessed their overall footprint (stain area normalized to cell diameter) using the protocol developed by Valente et al. 2017^32^. We found female neurons had a significantly larger footprint than males with TNFα treatment, (Fig. 4A–C,F(1, 14) = 4.782, P = 0.0462, Two-way ANOVA). This result prompted us to further investigate the specific morphological changes of these mitochondrial networks in response to TNFα treatment (Fig. 4D–G). We used a manual scoring method developed by Lebeau et al*.* 2018^33^. In male neurons, we observed a change in mitochondrial network morphology after 24 h of TNFα treatment compared to control, wherein the proportion of fragmented morphology decreased and the tubular morphology increased (P = 0.00062 and 0.0031 respectively, Fisher exact test, Bonferroni’s multiple comparison). However, female neurons exhibited changes in mitochondrial morphology at all three timepoints. At one hour of TNFα treatment, fragmented morphology increased, and tubular morphology decreased compared to control (P = 2.2e − 16 for both morphologies, Fisher exact test, Bonferroni’s multiple comparison). At 6 h and 24 h of TNFα treatment there was a shift in how the females responded to TNFα treatment. Like the male 24-h treatment, female neurons exhibited decreased fragmented morphology (P = 0.00012 and 3.3e − 11 respectively), and exhibited an increased tubular morphology compared to control (P = 0.000027 and 8.2e − 10 respectively, Fisher exact test, Bonferroni’s multiple comparison) at both 6 and 24 h of TNFα treatment. Next, we aimed to determine how these morphological changes related to more functional mitochondrial outcomes.

### Mitochondrial superoxide and cytochrome c release are increased in male sensory neurons with TNFα treatment in vitro

**Figure 5:**
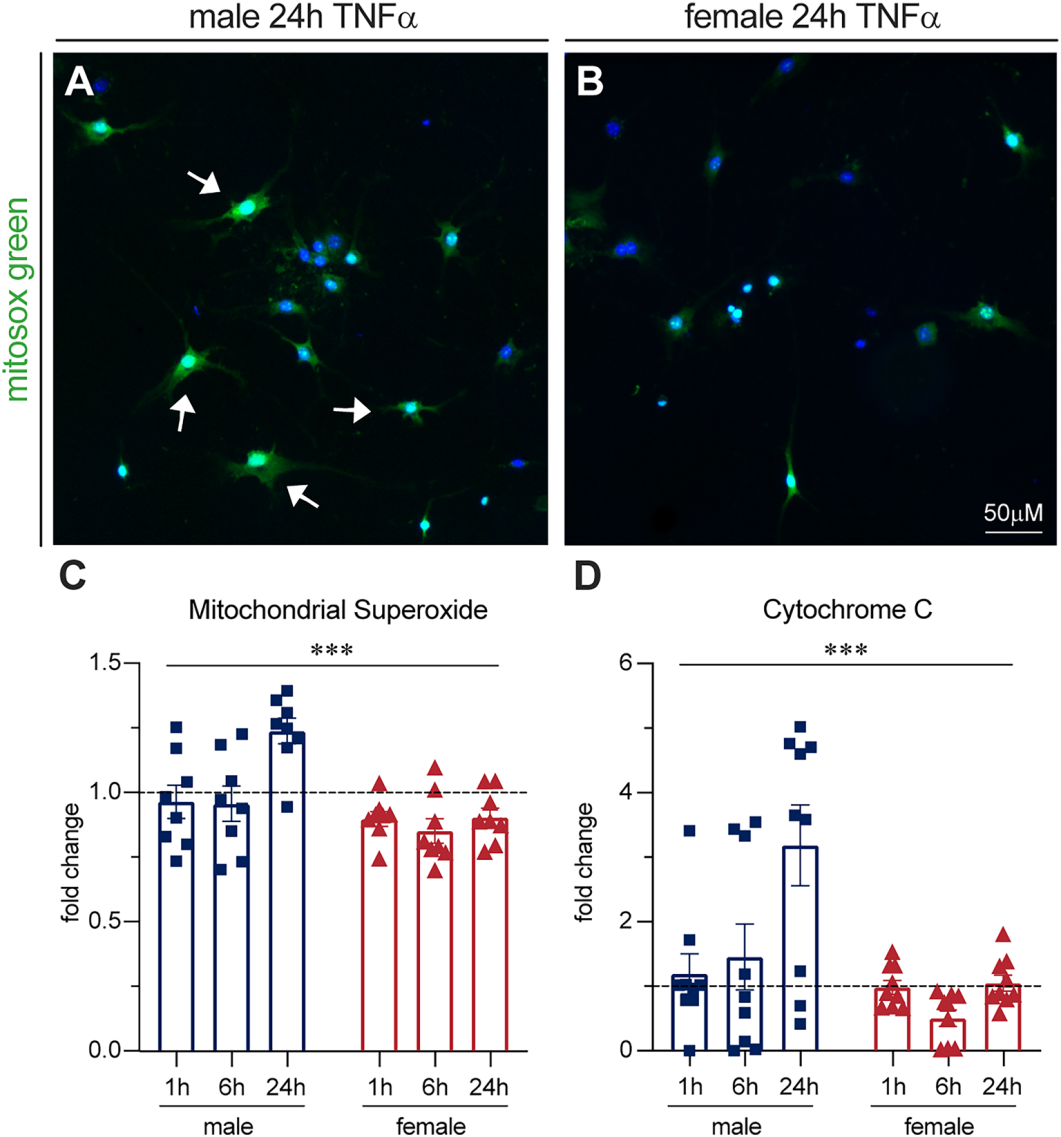
Increased mitochondrial superoxide and cytochrome C release in male cells with TNFα treatment in vitro. (**A,B**) Male DRGs increased live cell mitochondrial superoxide staining compared to females with TNFα treatment. Arrows indicate examples of positive staining. (**C**) Bars indicate mean (fold change of superoxide-positive cells/total DAPI-positive cells, normalized to untreated control) ± standard error mean (SEM). Dashed line represents untreated condition mean n = 8 across 2 experiments. ***p < 0.001, sex factor, two-way ANOVA. (**D**) Male DRGs increased cytochrome C release compared to females, measured by ELISA. Bars indicate mean (fold change of cytochrome C in ng/mL, normalized to untreated controls) ± standard error mean (SEM). Dashed line represents untreated condition mean n = 9 across 3 experiments. ***P < 0.001, sex factor, two-way ANOVA.

To determine the consequences of the mitochondrial changes we observed, we explored mitochondrial superoxide levels with MitoSOX™ live cell staining (Fig. 5A–C). There was significantly more superoxide staining in male cells in response to TNFα than female cells (F(1,42) = 16.40, P = 0.0002, Two-way ANOVA). To further understand the extent of male mitochondrial dysfunction we assessed levels of cytochrome c release from mitochondria in cell lysates by ELISA (Fig. 5D). We detected significantly more released cytochrome from male cells than female cells (F(1,48)-13.63, P = 0.0006, Two-way ANOVA). While both P38 and NFκB are linked to mitochondrial changes, P38 in particular can mediate intrinsic apoptosis through cytochrome c release^42^. We hypothesize that preferential activation of P38 in male cells may make them more susceptible to mitochondrial damage, which could cause neuronal damage more broadly.

### Male DRGs have more cleaved caspase 3 staining in vitro and in vivo

**Figure 6:**
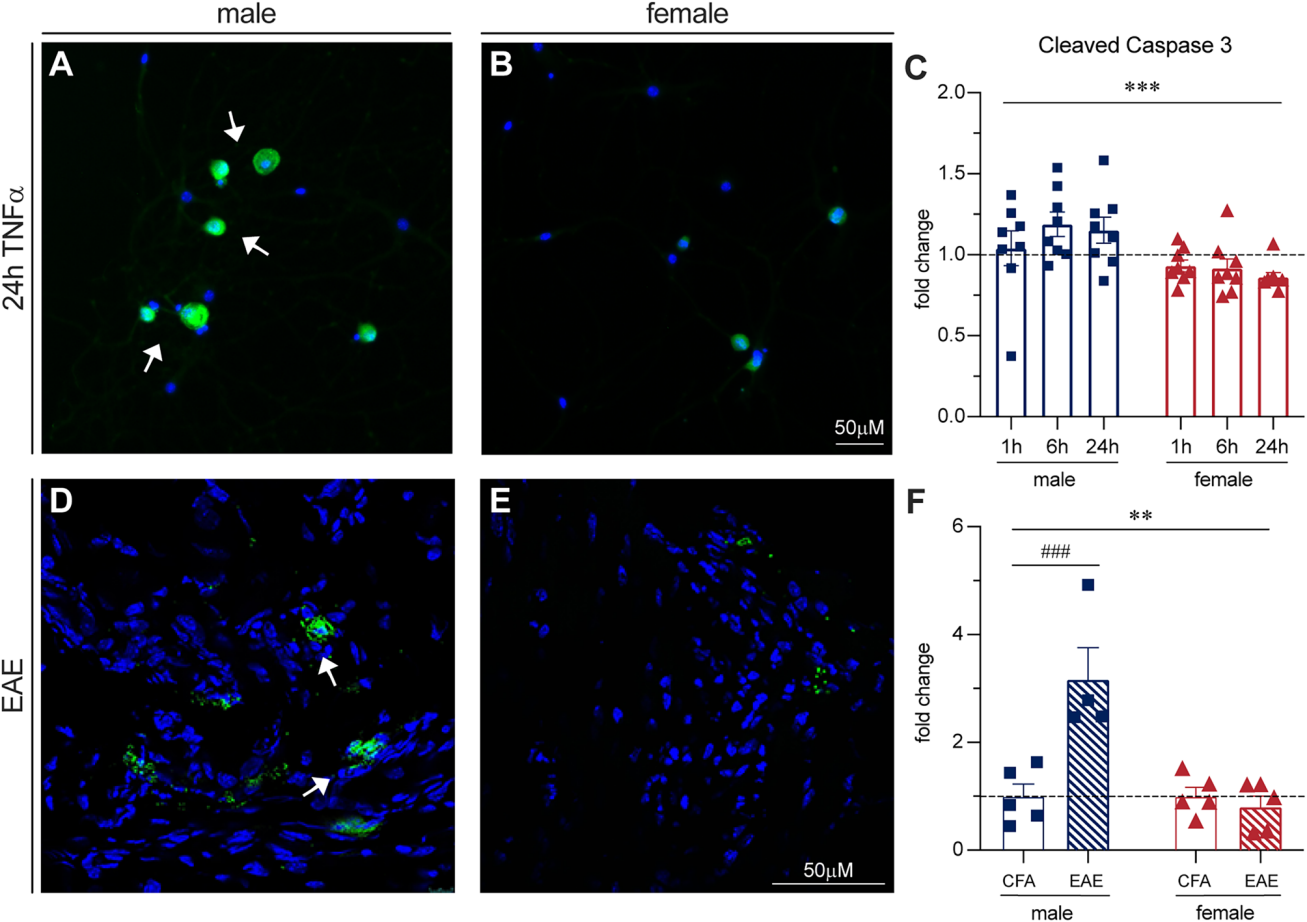
Cleaved caspase 3 staining increases in male DRGs both in vitro and in vivo. (**A,B**) Cleaved caspase 3 staining is increased in cultured male DRGs compared to females with TNFα treatment. Arrows indicate examples of positive staining. (**C**) Bars indicate mean (fold change of caspase-positive cells/ total DAPI-positive cells, normalized to untreated control) ± standard error mean (SEM). Dashed line represents untreated condition mean n = 8 across 2 experiments. ***P < 0.001, sex factor, two-way ANOVA. (**D–F**) Cleaved caspase 3 staining is increased in male EAE DRGs compared to females. Bars indicate mean fold change of positive cells normalized to DRG area) ± standard error mean (SEM). Dashed line represents untreated condition mean n = 5 animals in one experiment. ###P < 0.001 treatment factor, **P < 0.01, ***P < 0.001, sex factor, two-way ANOVA.

Cytochrome C release from mitochondria can lead to cell damage and eventual intrinsic apoptosis through apoptosome formation and activation of caspase 3 by cleavage from a pro- to active form^43^. Since we found increased P38 activation and cytochrome C release in male sensory neurons, we sought to determine whether this led to increased caspase 3 cleavage. Using immunocytochemistry to asses our TNFα-treated sensory neurons, we found there was significantly more cleaved caspase 3 staining in males than females (Fig. 6A–C,F(1,42) = 15.29, P = 0.0003, Two-way ANOVA). To validate our in vitro model, we next determined if this result occurred in vivo in EAE. In DRG sections from mice with EAE, there was indeed greater cleaved caspase 3 staining in males than in females (Fig. 6D–F,F(1, 15) = 14.97, P = 0.0015, Two-way ANOVA). These results suggest that male DRG sensory neuron activation of p38 in response to TNFα may trigger structural and functional mitochondrial changes, potentially resulting in increased neuronal damage through caspase 3.

### P38 inhibition reduces TNFα-induced cytochrome c release and caspase 3 cleavage in male cells in vitro

**Figure 7:**
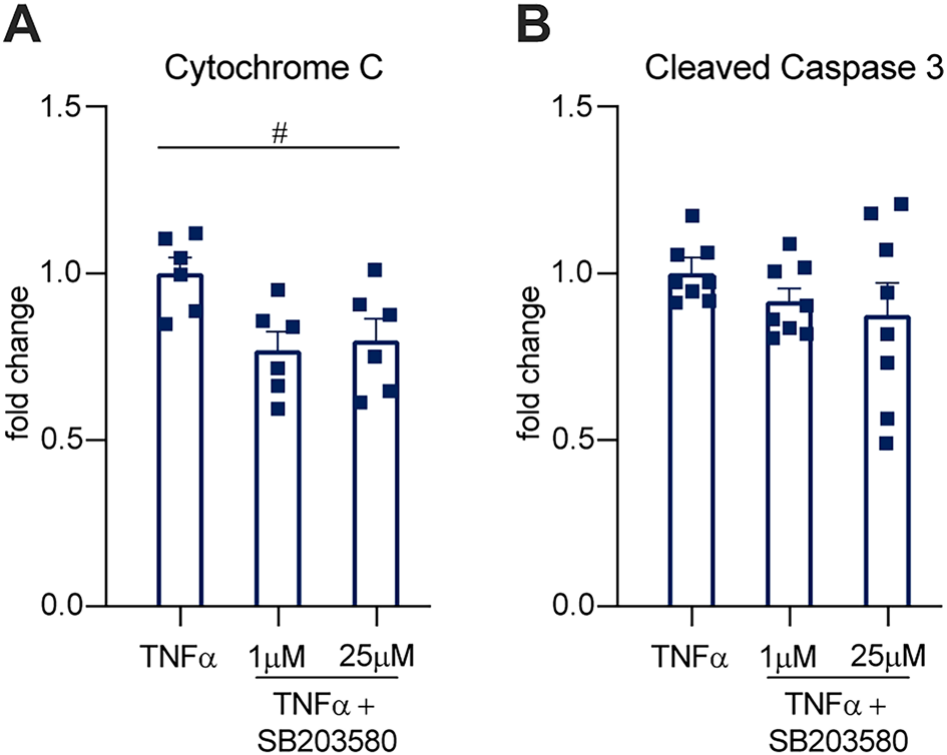
P38 inhibition reduces TNFα-induced cytochrome c release in vitro. (**A**) TNFα-induced cytochrome c release reduces with P38 inhibition by SB203580. Bars indicate mean (fold change of cytochrome C in ng/mL, normalized to TNFα treatment alone) ± standard error mean (SEM). Dashed line represents untreated condition mean n = 6 across 2 experiments. #P < 0.05, treatment factor, two-way ANOVA. (**B**) TNFα-induced cleaved caspase 3 staining is not significantly altered by P38 inhibition by SB203580. Bars indicate mean (fold change of caspase-positive cells/total DAPI-positive cells, normalized to TNFα treatment alone) ± standard error mean (SEM). Dashed line represents untreated condition mean n = 8 across 2 experiments.

To determine if P38 was the effector responsible for elevated cytochrome c release and caspase 3 cleavage in male cells we used a selective P38 inhibitor in the presence of TNFα stimulation. We treated cells with 1 ng/mL of TNFα for 24 h and no inhibitor, or a low (1 μM), or high (25 μM) dose of SB203580. We found that cytochrome c release, measured by ELISA, decreased with SB203580 treatment (Fig. 7A,F(2, 15) = 5.145, P = 0.0199, one-way ANOVA). Both the low and high doses significantly reduced cytochrome c release when directly compared to TNFα treatment alone (P = 0.0184 and 0.0390 respectively, Dunnett’s multiple comparisons). However, cleaved caspase 3 staining by immunocytochemistry was not significantly reduced (Fig. 7B,F(2, 21) = 1.068, P = 0.3618, one-way ANOVA). We hypothesize that this lack of significance despite a trend towards reduced cl-caspase 3 at the higher concentration of SB203580 may be due to assay sensitivity.

### Plasticity markers are increased in female EAE DRGs

**Figure 8:**
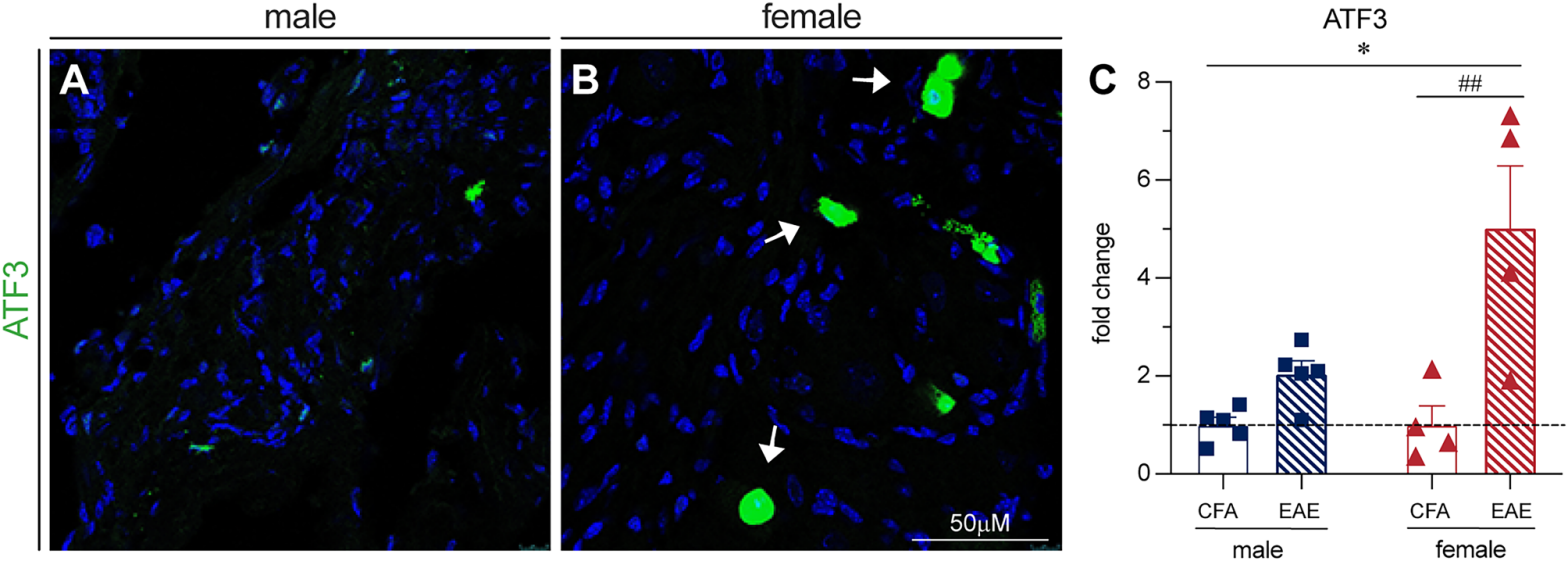
ATF3 staining increases in female EAE DRGs in vivo. (**A,B**) ATF3 staining is increased in female EAE DRGs compared to males. Arrows indicate examples of positive staining. (**C**) Bars indicate mean (fold change of ATF3-positive cells/DRG area, normalized to CFA control) ± standard error mean (SEM). Dashed line represents CFA condition mean. n = 5 animals in one experiment. ##P < 0.01 treatment factor, *P < 0.05 sex factor, two-way ANOVA.

**Figure 9:**
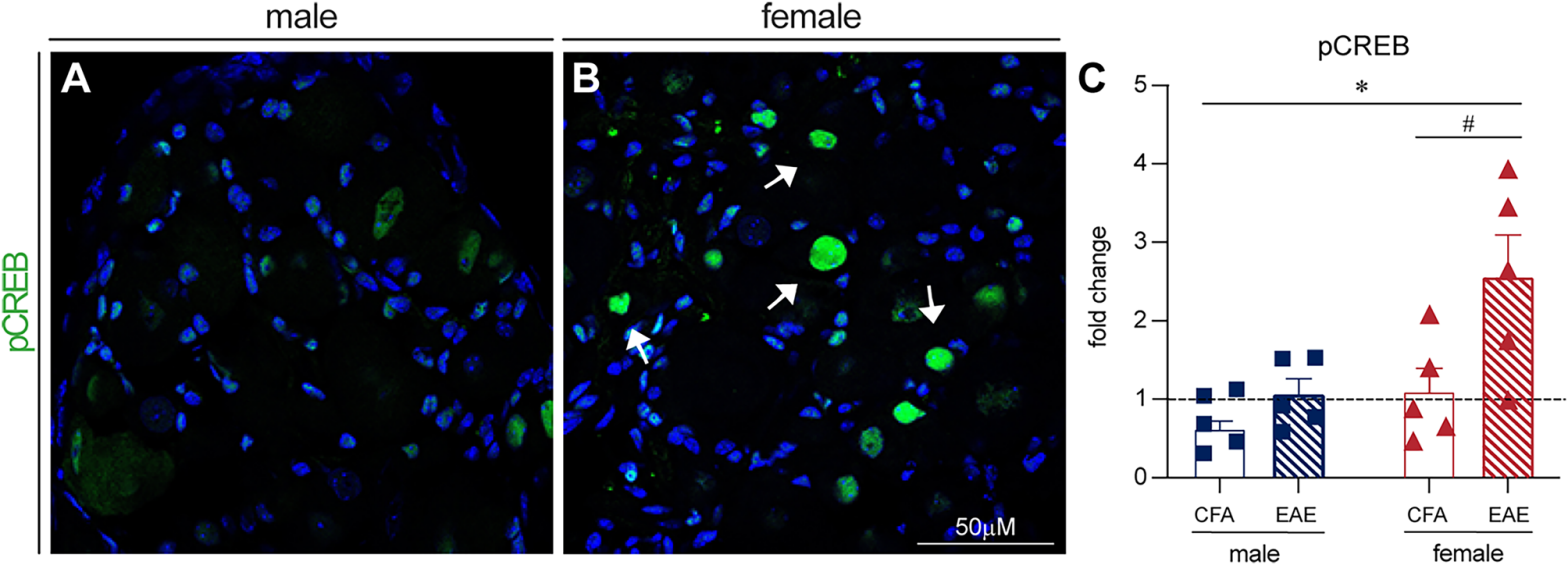
Phospho-CREB staining increases in female EAE DRGs in vivo. (**A,B**) pCREB staining is increased in female EAE DRGs compared to males. Arrows indicate examples of positive staining. (**C**) Bars indicate mean (fold change of pCREB-positive cells/DRG area, normalized to naïve control) ± standard error mean (SEM). Dashed line represents naïve condition mean. n = 5 animals in one experiment. #P < 0.05 treatment factor, *P < 0.05 sex factor, two-way ANOVA.

To gain an understanding of what mechanisms may be present in female EAE DRGs to prevent mitochondrial superoxide production, cytochrome C release, and caspase 3 cleavage, we investigated two plasticity-associated transcription factors. We first stained for activating transcription factor 3 (ATF3), an injury and plasticity-associated factor, in the DRG of mice with EAE (Fig. 8A–C). There was a significant increase in ATF3 staining in EAE DRGs compared to their CFA controls (F(1, 14) = 17.51, P = 0.0011, Two-way ANOVA, Sidak’s multiple comparisons test), with a significantly greater increase in females than males (F(1, 14) = 6.060, P = 0.0274, Two-way ANOVA). We also stained EAE DRGs for phosphorylated cAMP response element-binding protein (pCREB). CREB is a transcription factor strongly linked to neuroplasticity and pro-survival gene transcription as well as neuropathic pain^44,45^. We found a significant increase in pCREB staining in EAE DRGs compared to their CFA controls, normalized to naïve mice (Fig. 9A–C,F(1, 16) = 7.3, P = 0.0155, Two-way ANOVA), with a sex difference (F(1, 16) = 7.9, P = 0.0124, Two-Way ANOVA) where EAE females in particular demonstrated increased pCREB staining (P = 0.0135, Sidak’s multiple comparisons test). This female-dominated increase in ATF3 and pCREB staining in the EAE DRG suggests that females may have a stronger plastic and/or regenerative response to inflammation. This could explain their protection from mitochondrial dysfunction and neuronal damage, as well as contribute to their elevated hyperexcitability.

## Discussion

Our results reveal sex differences in the DRG in both the EAE mouse model of MS, and our TNFα-treated culture model. Both male and female EAE DRGs exhibit increased staining for immune cell markers, but notably, males have greater immune cell (CD45) and macrophage/monocyte (Iba1) staining than females at the onset of EAE. Using an in vitro preparation of DRG neurons stimulated with the inflammatory cytokine TNFα, we observed that males preferentially activated P38 MAPK, and presented with disrupted mitochondrial morphology, elevated mitochondrial superoxide staining, and cytochrome c release. We hypothesized that P38-mediated mitochondrial pathology may be responsible for the increased cytochrome c release in vitro and cleaved caspase 3 staining both in vitro and in vivo in male DRG neurons. We partially confirmed this hypothesis in vitro by showing that P38 inhibition reduces cytochrome c release in male DRG neurons. Females, however, preferentially activated the transcription factor NFκB rather than P38, and while their mitochondrial morphology was also altered, they did not exhibit elevated superoxide staining, cytochrome c release, or cleaved caspase 3 staining. Rather, we show that female EAE DRGs have increased staining for the plasticity-associated factors, ATF3 and pCREB in vivo. These observations may have significant implications for our understanding of pain in EAE animals.

First, we found a significant increase in inflammation and immune cell infiltration indicated by CD45, CD3, and Iba1 staining at the level of the DRG in the EAE model in both sexes. These findings align with previous work from our lab and others regarding the inflammatory response of peripheral ganglia in female EAE mice^15,34–36^. Many studies of neuropathic pain using peripheral nerve injury models have focused on the spinal cord and report that females have a T-cell-mediated pain phenotype^46,47^. This, however, may not be true for the onset of peripheral sensitization in the EAE DRG, as we saw no sex differences in CD3 T cell infiltration between the sexes. However, the indication that microglia/ macrophages are responsible for pain in the male spinal cord^46–49^ may be analogous to the EAE DRG, as evidenced by our finding that more cells were stained for CD45 and Iba1 in males. To our knowledge we are the first to report on a sex difference in EAE DRG inflammation. As such, future studies will be required to confirm this observation. DRG macrophages have been identified as important players in peripheral sensitization and neuropathic pain^50^. For example, their secretion of pro-inflammatory mediators, including TNFα, can increase sensory neuron excitability^51–53^. We found increased TNFα mRNA in both male and female EAE DRGs, and our lab has previously confirmed that TNFα is increased peripherally in EAE^12^. We used these findings to select a physiologically relevant dose of TNFα (1 ng/mL) and applied this stimulus to naïve primary DRG neuron cultures to further elucidate intracellular signaling sex differences in sensory neurons.

To determine how the immune profile that we identified in EAE DRGs might affect intracellular neuronal processes, we moved in vitro*,* where we observed sex differences in the downstream signaling pathways engaged by TNFα stimulation. Our finding that male neurons are biased towards activation of P38 in response to TNFα is supported by studies which have shown that males activate P38 in the spinal cord in mouse models of neuropathic pain^54,55^. Additionally, we observed that female neurons preferentially activate NFκB with TNFα stimulation. This aligns with reports indicating that females preferentially activate NFκB in endothelial cells and inflamed lung tissue^56,57^.

Before investigating the implications of P38 versus NFκB signaling between the sexes we assessed cell death by PI staining. Neurons express the TNF receptor isoform TNFR1, which mediates pro-inflammatory signaling as well as programmed cell death^40^. We found increased cell death with TNFα treatment in DRG neurons compared to untreated controls, particularly with 24 h of TNFα treatment. TNFα signaling can lead to cell death through either necroptosis or intrinsic apoptosis^40^. We decided to investigate the possibility of intrinsic apoptosis, which involves mitochondria damage and cytochrome c release. Given that both P38 and NFκB are linked to functional changes in mitochondria^24–27^ this seemed like a promising avenue of study.

We went on to first conduct a surface-level investigation of the changes that occur in mitochondrial morphology in response to TNFα. We found that female had a greater mitochondrial footprint than males in response to TNFα treatment, and that their mitochondrial morphology changed more in the treatment timeline than males. We observed more fragmented mitochondria, and less tubular mitochondria in females after one hour of TNFα treatment compared to control, which may indicate a maladaptive phenotype such as elevated mitochondrial fission. However, at 6 and 24 h of TNFα treatment, female mitochondrial networks appeared to shift their response to TNFα treatment, as they exhibited less fragmented mitochondria and more tubular mitochondria compared to control. Male neurons also exhibited less fragmentation and more tubular networks at 24 h of treatment but showed no significant changes at earlier timepoints.

To further investigate mitochondrial health in our TNFα-stimulated culture, we turned to the more functional readouts of superoxide production and cytochrome C release. We found that male DRG neurons had more staining for mitochondrial superoxide and more cytochrome C release than females in response to TNFα. We hypothesize that the differential activation of the TNFα signaling pathway in male and female DRG neurons might be responsible for these functional differences, and that males might be more prone to neuronal damage as a result.

The dramatic increase in cytochrome C release from male neurons lead us to investigate caspase 3 cleavage as the next consequence of the intrinsic apoptotic pathway. We found that both in vitro and in vivo, male DRGs exhibit more cleaved caspase 3 staining than females. This finding aligns with the TNFα signaling literature as P38 can cause cytochrome C release and eventually, activate intrinsic apoptosis through B-cell lymphoma (Bcl) protein regulation^42^. Previous work from our lab on the spinal cord in EAE aligns with the theory that males may be more prone to damage, as we have found significantly greater increases in the levels of amyloid precursor protein (APP) and non-phosphorylated neurofilament (SMI32) in males, suggesting that male spinal neurons are more sensitive to axonal injury and degeneration in EAE^58^. Neuronal injury in males may also explain previous work from our lab which has shown that while female EAE DRG neurons are hyperexcitable, DRG neurons from male mice with EAE are not^12^.

To test the link between P38 and intrinsic apoptosis we treated male naïve cultured DRG neurons with the P38 inhibitor SB203580 at a low and high dose. Both doses reduced TNFα-induced cytochrome c release measured by ELISA. While cleaved caspase 3 staining also appeared to trend down with P38 inhibition, this data was not statistically significant. We hypothesize that because we only detected a small change in cleaved caspase 3 staining with TNFα treatment to begin with, the assay may not be sensitive enough to detect further changes with inhibition. Nevertheless, P38 inhibition in EAE animals is an avenue for future study to confirm this relationship.

Finally, we wondered why female neurons did not exhibit increased mitochondrial superoxide staining, cytochrome C release, and cleaved caspase 3 staining. We hypothesized that they might engage pathways linked with neural plasticity that prevent mitochondrial dysfunction and neuropathy, so we examined mouse EAE DRGs for the expression of the plasticity-associated proteins ATF3 and pCREB^59–61^. Females had significantly more ATF3-positive cells than males, suggesting that females more robustly engage this adaptive mechanism in response to inflammation. Interestingly, NFκB is known to induce ATF3 signaling, acting in a negative feedback loop to dampen its own pro-inflammatory signaling, as ATF3 can bind and inhibit the P65 subunit of NFκB^62–64^. There was also significantly more pCREB staining in the female EAE DRG than in males, which provides further evidence that the female DRGs are more likely to engage pathways linked with neural plasticity. Much like ATF3, pCREB can directly inhibit NFκB and pro-inflammatory signaling^65^. Importantly however, it has also been linked to aberrant plasticity, neuronal hyperexcitability, and pain in spinal cord injury^44,45^. While activation of these transcription factors in the female EAE DRG may protect neurons from mitochondrial pathology and apoptosis, they may also be responsible for maladaptive plasticity, leading to neuronal hyperexcitability. This link, as well as the mechanism of cell death in females will be investigated further in future studies.

Taken together, we propose that male DRG neurons preferentially activate P38 in response to TNFα, which may lead to mitochondrial dysfunction, cytochrome c release and caspase 3 cleavage (Fig. 10.1). This pathway may cause male EAE DRGs to become more prone to neuropathy and apoptosis, and male pain in EAE might be mediated by a central rather than peripheral mechanism. We have provided evidence for the relationship between P38 and cytochrome c release by inhibiting P38, but this pathway will require further investigation in our future work. In females, a regenerative mechanism may be induced by preferential NFκB signaling in DRG neurons in response to TNFα (Fig. 10.2). While this mechanism could protect against apoptosis, it may allow for cell death through other means, and may even contribute to neuronal hyperexcitability and pain in EAE through aberrant plasticity^66,67^. The relationship between mitochondrial changes, cell death, plasticity, and pain in females will require further investigation in future studies. Taken together, these studies reveal that there are distinct pathological changes that occur at the level of the DRG between the sexes. There is a need for future therapeutic strategies to treat neuropathic pain to be tailored in a sex specific manner. Our findings suggest that targeting different neuronal pathways in the DRG of male and female EAE animals could aid in the development of these new treatments.

**Figure 10:**
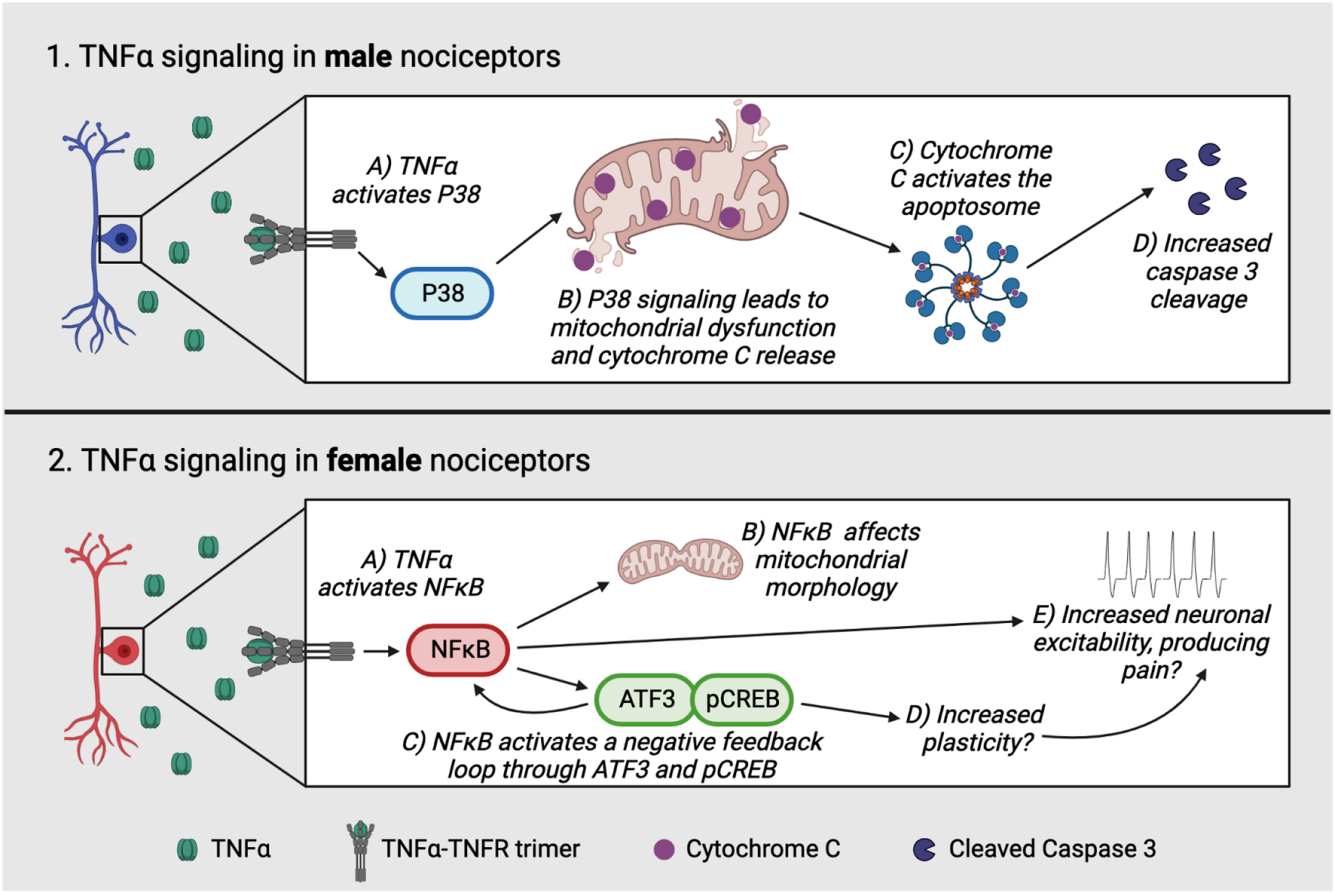
Working hypothesis of sex differences in TNFα signaling. (1**A-D**) In male DRG neurons, we hypothesize that TNFα activates P38 signaling, triggering mitcohdondrial dysfunction, cytochrome c release, and caspase 3 cleavage, which may contribute to neuropathy and degeneration. (2**A–C**) In female DRGs, we hypothesize that inflammatory TNFα signaling activates NFκB, which affects mitochondrial morphology, activates ATF3 and pCREB, and may contribute to pain. (2**D,E**) ATF3 is known to negatively regulate NFκB, and is also associated with plasticity which may also contribute to pain.

## Supplementary Information

Supplementary Figure 1.

Supplementary Figure 2.

## Data Availability

Applicable data and materials available upon reasonable request. Please contact Bradley Kerr at bradley.kerr@ualberta.ca.
