# Supplementary figures and images for: Sex differences in the inflammatory response of the mouse DRG and its connection to pain in experimental autoimmune encephalomyelitis
Source: Sci Rep. 2022 Dec 5;12:20995. doi: 10.1038/s41598-022-25295-y (PMC9722825; doi:10.1038/s41598-022-25295-y)

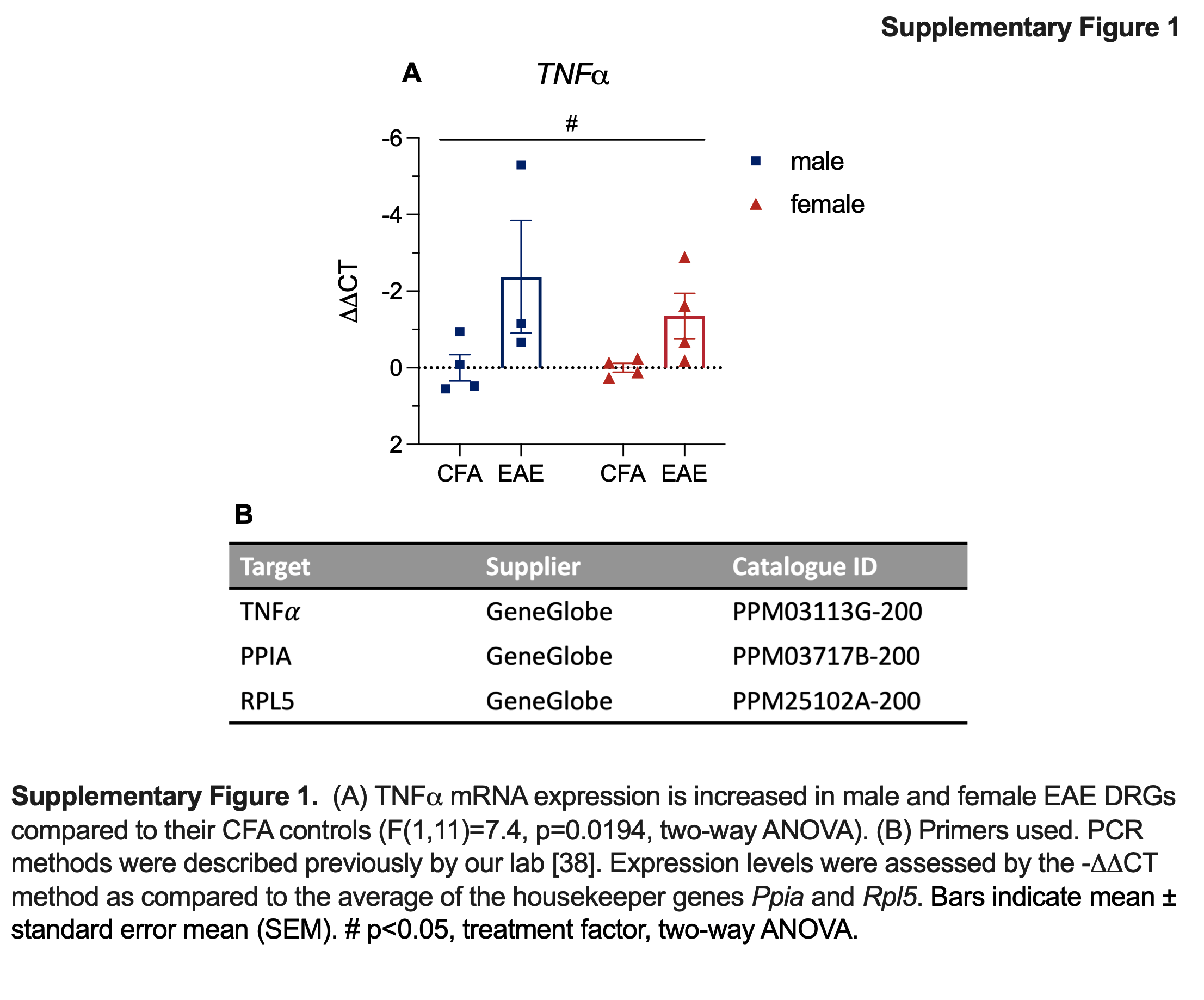

Supplement: Supplementary file 1 — Supplementary Figure 1. [file 41598_2022_25295_MOESM1_ESM.tiff]

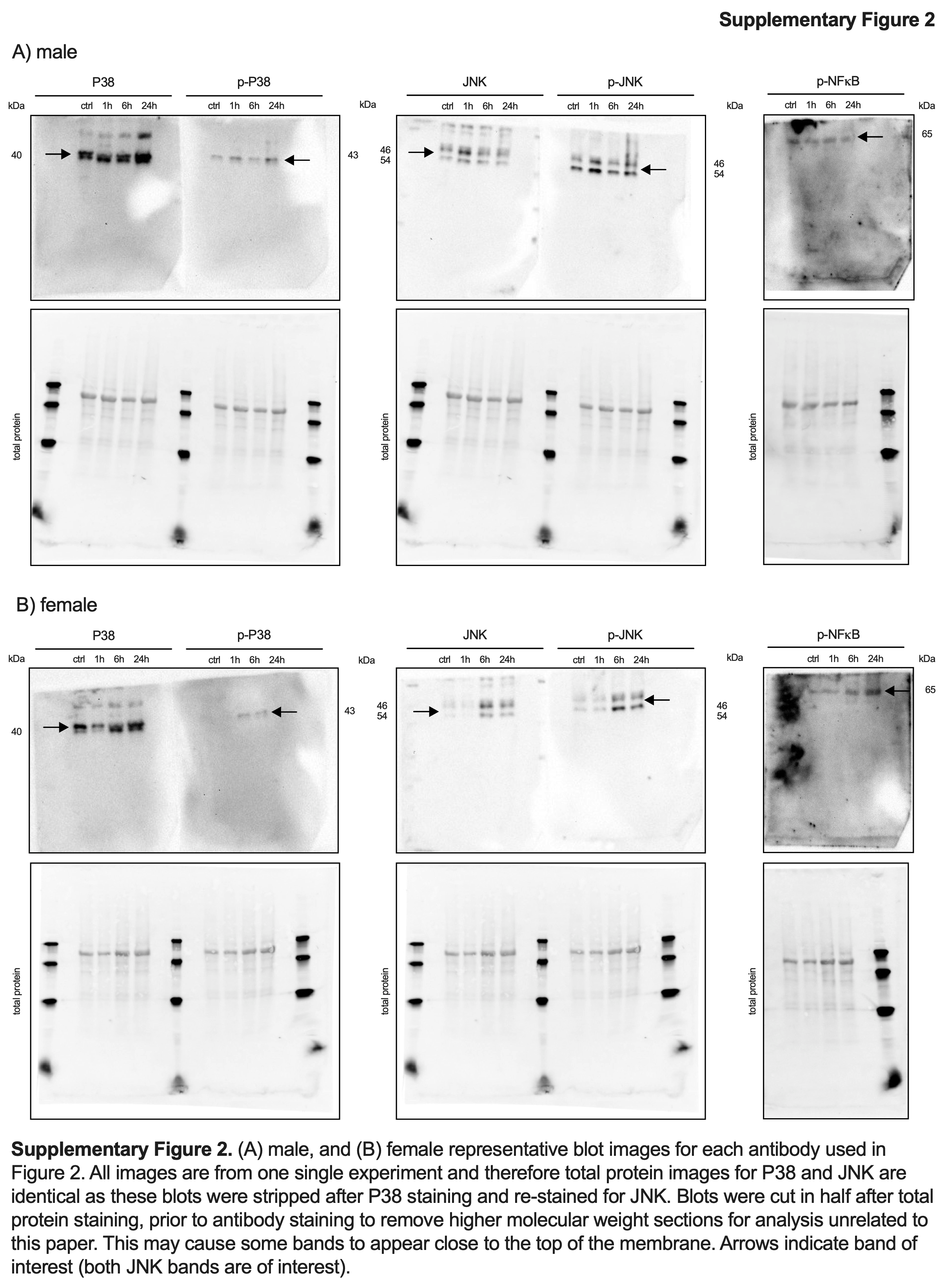

Supplement: Supplementary file 2 — Supplementary Figure 2. [file 41598_2022_25295_MOESM2_ESM.tiff]
